# Lethal means and suicide prevention among military veterans

**DOI:** 10.1186/s40779-022-00400-4

**Published:** 2022-07-05

**Authors:** Joshua Levine, Leo Sher

**Affiliations:** 1 Veterans’ Administration New York Harbor Healthcare System, Brooklyn, NY 11209 USA; 2 James J. Peters Veterans’ Administration Medical Center, Bronx, NY 10468 USA

**Keywords:** Suicide, Depression, Public health, Lethal means, Military veterans

Dear Editor,

Suicide amongst the military veteran population is a significant public health problem in the United States. The National Veteran Suicide Prevention Annual Report revealed that 6261 died by suicide in 2019 [1]. The lingering effects of the coronavirus disease 2019 (COVID-19) pandemic may account for an increase in veteran suicide rates [1]. Lethal means play a consequential role in veteran suicide deaths. Restriction of access to lethal means should be an important focus of suicide prevention among military veterans.

In 2019, firearms accounted for 69.2% of veteran suicides, followed by 19.9% suffocation, 8.4% poisoning, and 5.4% other methods (i.e., jumping, cutting, drowning) [1]. Firearms are especially concerning due to the veteran’s knowledge of firearms and military experience [1, 2]. Veterans who have access to firearms should be provided with gun locks/lockers, and encouraged to store their ammunition away from their firearms [2]. Medical practitioners must take a more vigilant approach to veterans who own firearms and have a psychiatric diagnosis by conducting regular suicide screens.

Limiting access to over-the-counter medications is another important area of means restriction to consider [3]. Veterans with suicidal tendencies should not be given large quantities of prescribed medications, especially medications that can be lethal (i.e., lithium). The veteran’s healthcare providers should actively communicate and collaborate on treatment to prevent superfluous prescribing. Veterans can be given medication lockboxes and pill holders to safely store their medications. A trusted family member could retain the key to the medication lockbox, and safely dispense and monitor the amount of tablets.

Lethal means restriction is effective in preventing suicides [1, 4]. Studies have shown a reduction in suicide attempts and deaths by increasing time and distance between the chosen suicide method and the actual suicide attempt [4]. The suicide safety plan is one tool that can be utilized to assess access to lethal means, and provide a detailed written plan on how to restrict access to lethal means [5]. The suicide safety plan should be completed in collaboration with the veteran, and consider all types of lethal means [5]. The veteran should be offered the opportunity to include family members in the safety plan meeting, so they can better support the veteran and be educated on firearm safety and safe medication use.

Medical practitioners who treat military veterans must make a conscious effort to regularly conduct suicide screens and assess for lethal means. Restricting access to lethal means such as firearms and medications is an effective way to mitigate suicide risk, and should be a focus of suicide prevention interventions in the military veteran population.

## Data Availability

Not applicable.

## Abbreviation

COVID-19: Coronavirus disease 2019
